# Supplementary material for: Cyclic Stretch-Induced Mechanical Stress Applied at 1 Hz Frequency Can Alter the Metastatic Potential Properties of SAOS-2 Osteosarcoma Cells
Source: Int J Mol Sci. 2023 Apr 22;24(9):7686. doi: 10.3390/ijms24097686 (PMC10178551; doi:10.3390/ijms24097686)

Supplementary materials

Selection of the mechanical stretching regimen for SAOS-2 osteosarcoma cell line

Since during fast walking activities, bone cells are subjected to moderate elongation (Papachroni et al. 2009), we hypothesized that cells might evolve a mechanobiological response to a physiological stretch. As happens to any type of material, cells, when subjected to an external mechanical force, also undergo strain (structural deformation), which can vary in magnitude and mode depending on the intensity and the frequency of the applied force (Alloisio et al. 2021). As a matter of fact, cell response to physical cues (i.e. mechanobiology) is related not only to structural deformation (i.e. biomechanics), which takes place at the molecular level but more importantly to the number of applied cycles per second (strain frequency) (Jung et al. 2015, Zhou et al. 2021). Interestingly, cell response to physical cues (i.e. mechanobiology) is related not only to structural deformation (i.e. biomechanics), which takes place at the molecular level but more importantly to the number of applied cycles per second (strain frequency) (Jung et al. 2015, Zhou et al. 2021).

Current data demonstrate that a broader cell biology response to mechanical signals seems to correlate with an increased frequency, meaning that minor strains induced by lower forces applied more frequently are ample to stimulate bone formation (Alloisio et al. 2021).

Therefore, our culturing experimental model was stimulated with a medium magnitude strain; specifically, a magnitude of 4830 με elongations (corresponding to 0.5% elongation) was chosen because this value is slightly lower than the threshold of 5000 με beyond which bone tissue damage has been reported (Papachroni et al. 2009). We started the investigation by screening the best frequency for stretch stimulation able to induce the widest biological response on SAOS-2 cells. *Ab initio* cell behaviors of SAOS-2 cells were seeded either on silicone support or conventional plastic supports at the same cell density (150 cells/m^2). After three days, the nicotinamide adenine dinucleotide phosphate (NADPH)- dependent cellular oxidoreductase activity of cellular enzymes recorded by the MTT assay for those cells cultured on silicone plate was found to have a 1.77-fold-decrease compared to those grown under conventional tissue culturing plastic plate ($p<0.01$, Figure **S1 A**). This significant drop in cell viability came as no surprise because it is likely that a less stiffened plate does not provide the physiological environmental forces required for the anabolic stimulation of osteoblastic cells (Nagaraja et al., 2014). Interestingly, a proper mechanical stimulation (i.e.: 0.5% elongation, 1Hz) busted cell proliferation at three days. Specifically, for 3 days, SAOS-2 cells on silicone were exposed to one of three specific stretching regimens applied cyclically (for 1 hour every 3 hours of rest): i) high strain 25116 με (2.5% ε), at the 0.2 Hz rate frequency, ii) medium strain 4830 με (0.5%ε), at 1 Hz rate frequency, iii) low strain 2000 με (0. 2% ε) at the specified rate frequency 2.5 Hz. Supplementary figure **S1 A** shows that the medium strain 4830 με (0.5%ε) applied at 1 Hz rate frequency was the sole stretching regimen capable of boosting the viability of SAOS-2 cells: the MTT assay was found to have a 1.33-fold-increase compared to its unstretched counterpart ($p<0.001$) (Fig. **S1 A**). Given the distinguished biological response to the 0.5% elongation applied at 1 Hz frequency, this specific stimulation has been considered a valuable insult to understanding important cellular aspects of the behavior of osteosarcoma cells. However, we believed that after three days of culturing SAOS-2 cells on silicone, the difference we observed on stretched cells likely corresponds to multiple overlapping molecular scenarios (e.g., unpaired cell cycles), which could mask the understanding of the

mechanisms of cell mechano-response to cyclic stretching. Therefore, to simplify the understanding of the mechanobiological response, we decided to minimize the mechanical-induced changes by investigating the effect of a shortened cell exposition on the selected stimulation. As supplementary figure **S1 B** shows, the cell vitality at 24 hours of cells cultured on silicone (both static and stretched) resulted not dissimilar, from conventional tissue culturing plastic substrate. Therefore, all the experiment simulations were performed for 24 hours under these selected conditions (i.e.: 0.5% elongation applied at 1 Hz cyclical frequency), as within this time frame SAOS-2 cell metabolism is not dependent on the material of the plate.

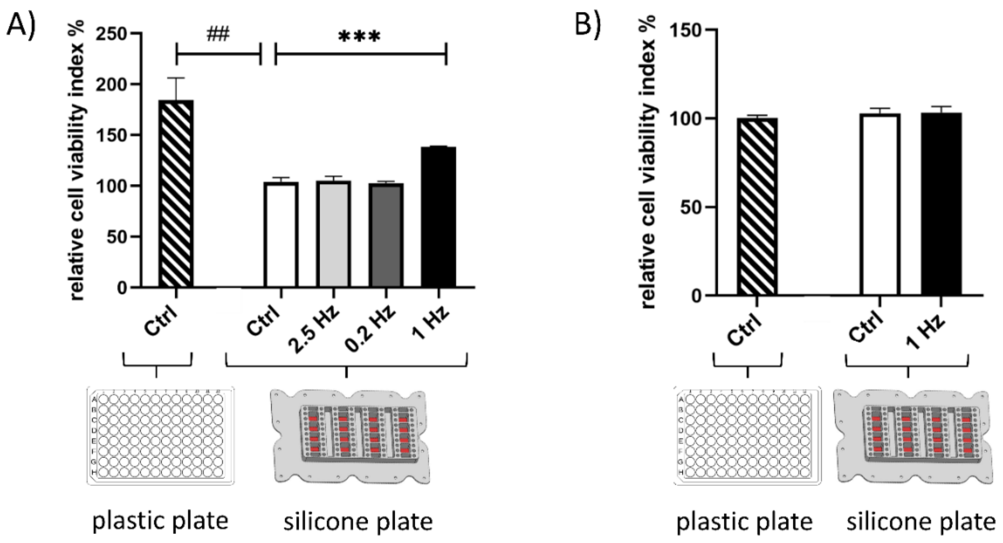

Figure S1. Screening of the mechanical stress conditions capable of inducing a specific cellular mechano-response in SAOS-2 osteosarcoma cells. **A**, Cell viability following 0.5% elongation applied for three days at different cyclic frequencies (2.5 Hz, 1 Hz, and 0.2 Hz); Ctrl refers to static cultures (these cells did not undergo any stretching). **B**, Cell viability of cells treated or not with 1 Hz frequency cyclic stimulation for 24 hours. Student's t-test was used for statistical analysis, and the results are shown as the mean ± SD. ***p<0.001, ## p<0.01, treated cells compared with the control cells. Statistic has been performed on three biological replicates with at least three technical replicates per condition.

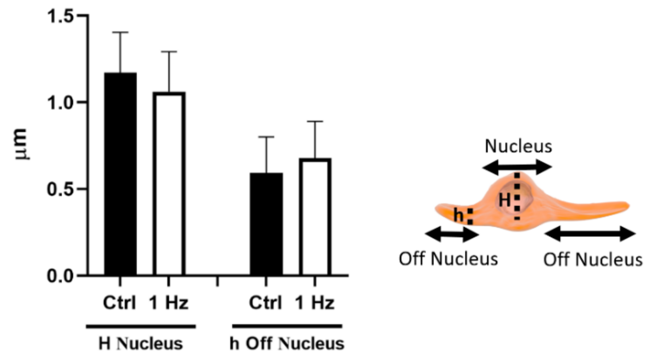

Figure S2. AFM analysis of the height of SAOS-2 cells fixed on glass. The graph illustrates the morphological changes induced in the nucleus and off-nucleus region by the pretreatment of a 24h-1Hz-uniaxial cyclic stretch of SAOS-2 cells. The cell drawing displays black the regions where the measurements were taken (i.e.: either the cell nucleus or off-nucleus areas (https://smart.servier.com). The column bar graphs report plot means and SD of the heights of the nuclei and off-nucleus regions and display the difference in the means of cyclically stretched (white histograms) and unstimulated (black histograms). A paired Student's t-test was used for statistical analysis. The statistic has been performed on three biological replicates with at least thirty-five measures per condition.

Random orientation of elongated cells

The long axis of our mechanically-treated cells did not display any preferential orientation (Fig. **S3**). As far as we know, the first evidence that osteoblastic cells subjected to deformations reorganize their actin cytoskeleton and align their long axis in the direction of the minimal substrate deformation, with a perpendicular orientation with respect to the force applied was reported by Boccafoschi et al 2010. In that paper, MC3T3-E1 osteoblasts underwent cyclic stretched 4%, at 1 Hz, for 3, 4, and 7 days of the cyclic stretch stimulation. Since our stimulation is less intense and shorter, the random orientation of cells comes as no surprise.

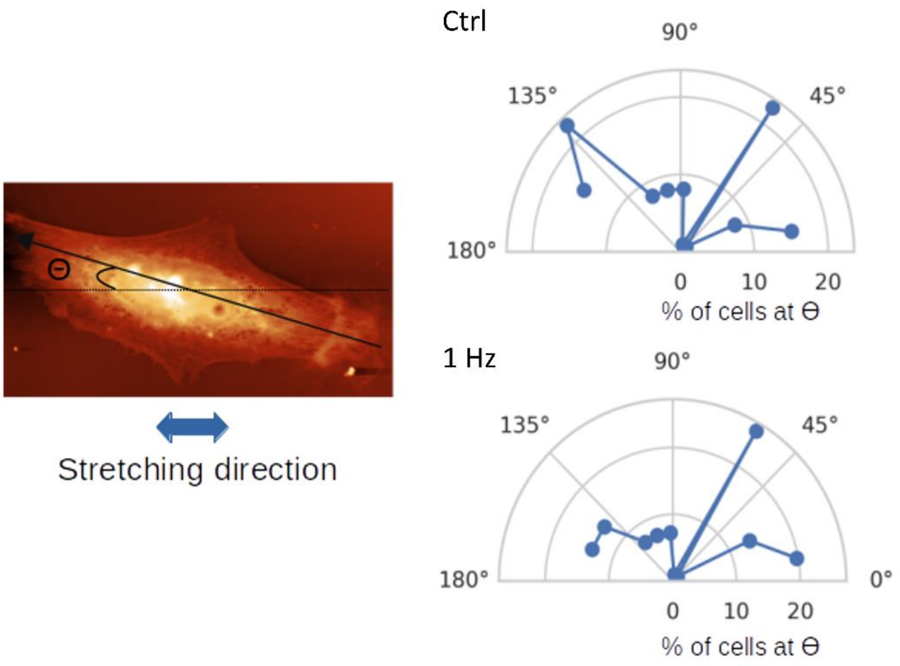

Figure S3. Statistics on the cell orientation of the control and 1 Hz samples with respect to the x-axis of stretching direction. The orientation was calculated by measuring the angle between the stretching direction (x-axis in the AFM images) and the cell major axis as shown by Θ in the AFM picture. The radial histograms describe the percent of the cells of each sample found at Θ±9°, effectively dividing the 180° into 10 bins.

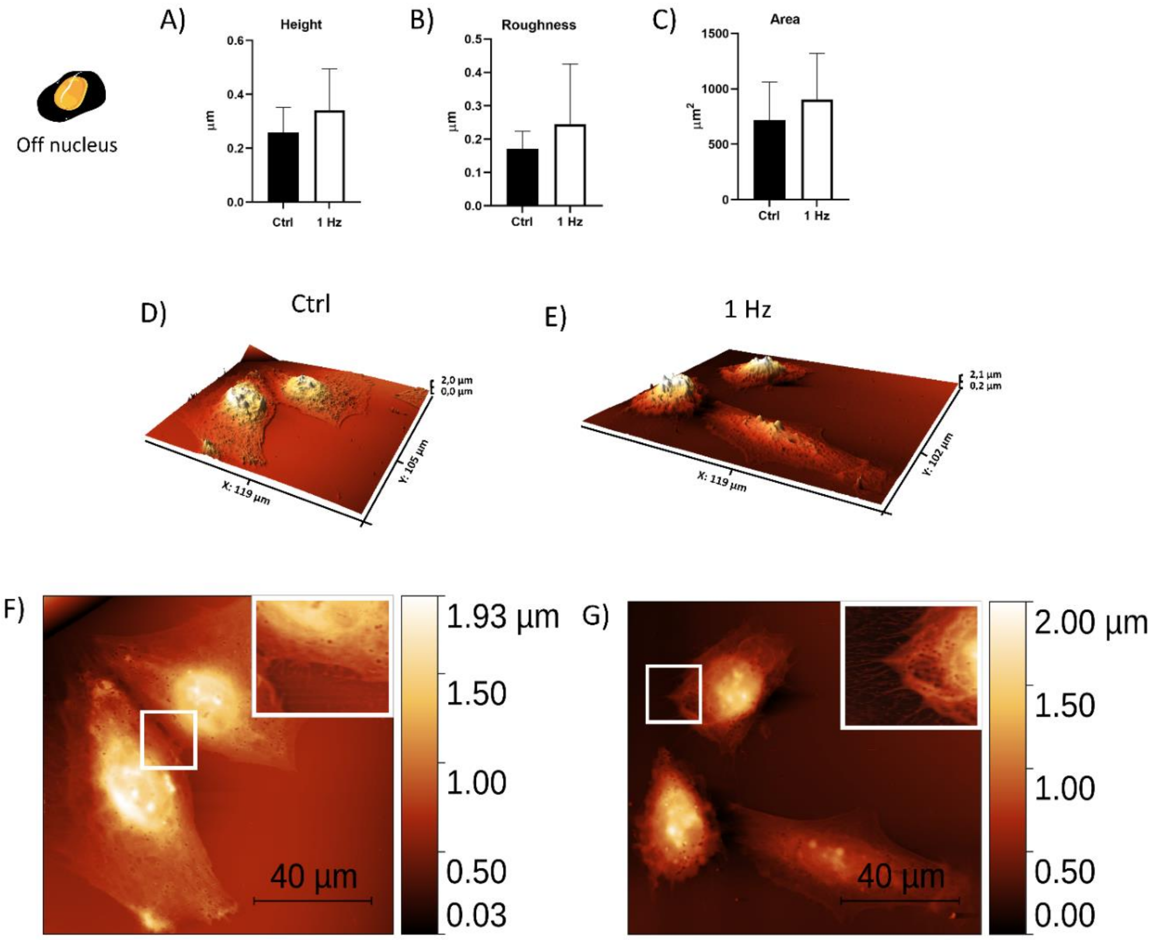

Figure S4. AFM analysis of the off-nucleus areas*.* The cell drawing displays black the regions where the measurements were taken (i.e.: off-nucleus region). Panels **A B** and **C** report the means ± SD of the measures for height, roughness, and area. Student's t-test was used for statistical analysis, and the results are shown as the mean ± SD. Statistic has been performed on 15 biological replicates with at least three technical replicates per condition. Panel **D** and **E** report 3-D and 2-D projections of representative AFM images cell boundaries of untreated and treated SAOS-2 cells (approximately 100 x100 µm with enhanced contrast). Panel **F** and **G** display a zoom of the cell peripheral areas allowing for a qualitative assessment of the difference in cell boundaries for control and treated cells.

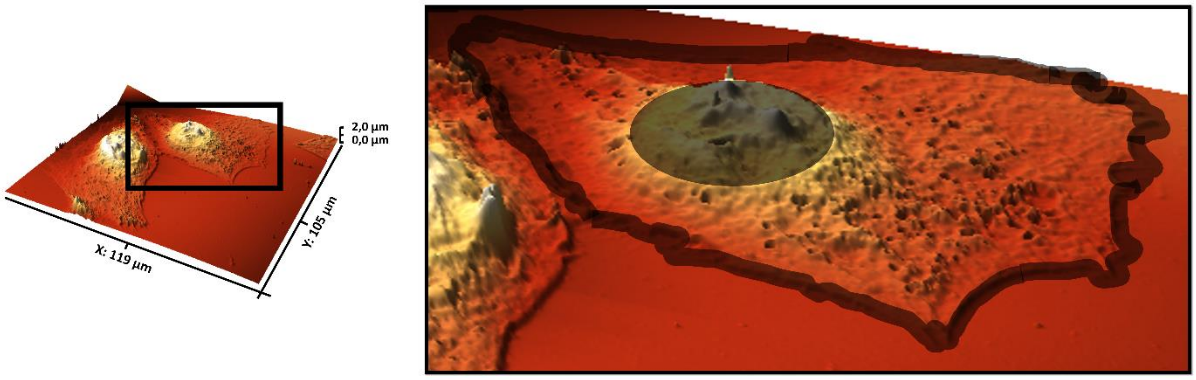

Figure S5. A portion of the 3-D and 2-D projections of supplementary Fig S4 was enlarged to show the regions where the AFM measures were taken). The gray shadow highlights the portions of the cell where the analyses were restricted to. (:i.e. the nucleus (Fig.1) and the cell periphery (Fig.3).

Sketch of the methodological experiments

The measurements reported in Figs 1,3, S3, S4 (A-C), and 4(A-C) were performed on a silicone plate. While in Figs 4D, 5 and 7 right after the mechanical treatment, the stretched cells and their static control counterparts were moved to conventional support. Going into the details, cells were trypsin detached, pelleted, and processed according to the procedures described in the materials and methods section (supplementary Fig **S7**).

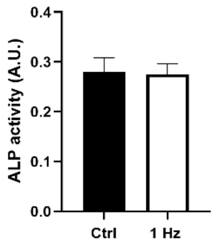

Figure S6. Cell-based ALP assay on a 96-well plate of adherent SAOS-2 cells using fluorogenic peptide (p-Nitrophenyl Phosphate (PNPP)). The 1 Hz 24 h stretch stimulation did not impact the osteoblastic properties of SAOS-2 cells. The comparison between measurements displays black histograms for untreated cells and white histograms for 1 Hz 24 h treated cells (error bars represent the standard error of the mean). Student's *t*-test of the histograms of Panel showed no significant differences between the treated cells compared with the control static cells. Statistical analyses were performed on three biological replicates with at least three technical replicates per condition.

On a 96-well- plate ALP assay

The stretched samples and control counterparts were processed immediately after the mechanical treatment, as follows: the impact of the mechanical stimulation on the specialized metabolism of the osteoblast was

observed by following the enzymatic activity of alkaline phosphatase and employing 4-NPP (p-nitrophenyl phosphate) colorogenic substrate, which becomes p-nitrophenolate after ALP enzymatic processing (normalized for 1 microgram of cell proteome). The phosphate activity of the ALP enzyme extracted from cell pellets with 0.1% NaCl, 0.2% Tween-20 lysis buffer was measured in 200 mM Tris, 2 mM $MgCl_2$, 0.05mM $ZnCl_2$ pH 8, with 20 mM 4-nitrophenyl phosphate (4-NPP) as a substrate. Alkaline phosphatase activity was measured by exploiting the hydrolytic capacity of a 1 µg cell extract to convert p-nitrophenyl sulfate to p-nitrophenol and phosphate. The colorimetric reaction, assessed by measuring the absorbance of the product at 405 nm, is directly proportional to the activity of the alkaline phosphatase contained in the sample. The linear increase of the optical density at 405 nm over time (up to 30 minutes) was measured with a TECAN spark^{R} multimode reader (Tecan group Ltd., Männedorf Switzerland) at 37°C .

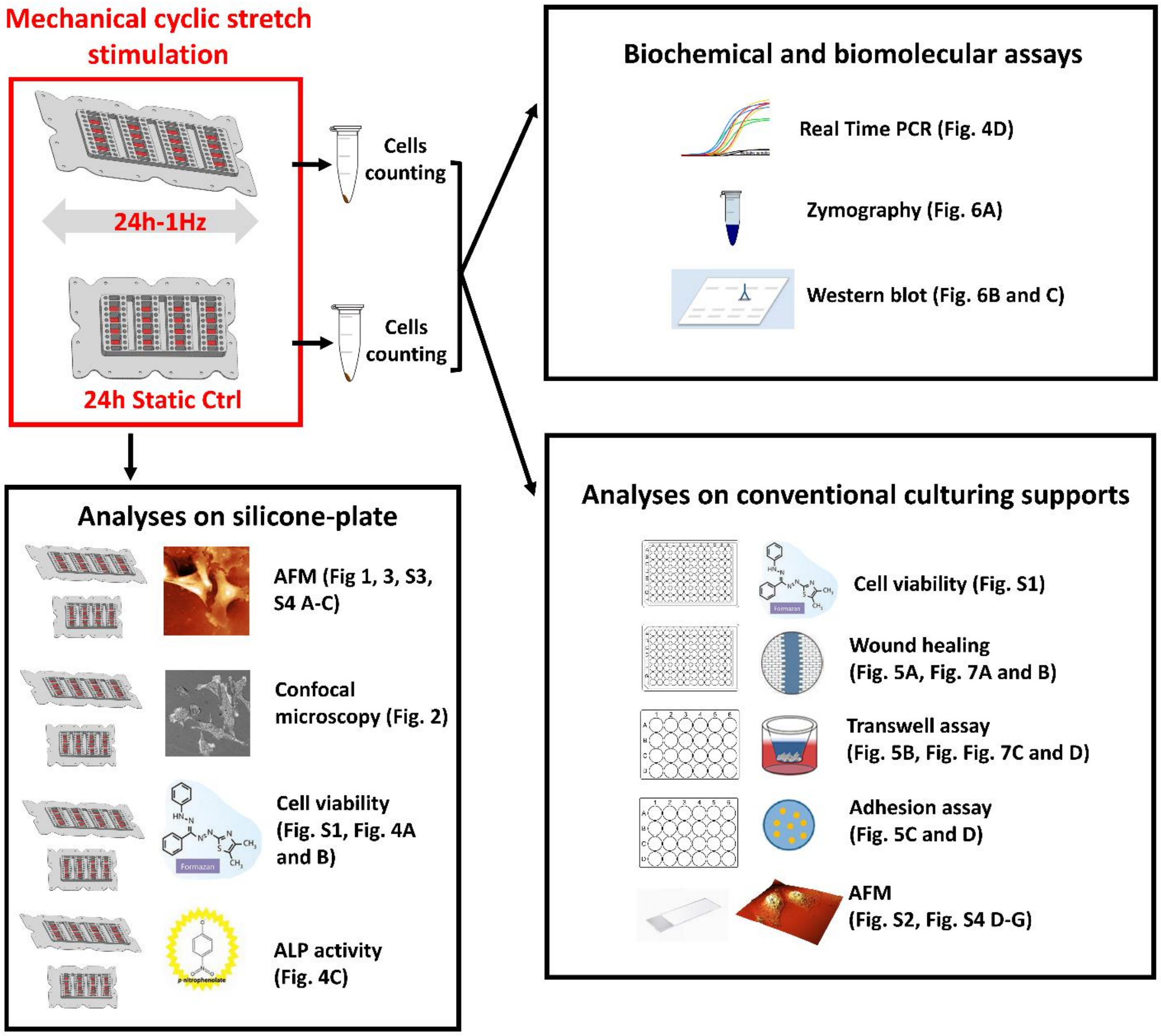

Figure S7. Graphical representation of the methodological setting used for each analysis reported by the figures of the manuscript.

Setting up of the MechanoCulture FX (MCFX) device for real-time absorbance and fluorescence readings

MCFX device allows for high throughput uniaxial stimulation of cell cultures with real-time imaging (Tran et al, 2021). A list of the publications where cell scale devices were employed can be found at the following link https://www.cellscale.com/publications/

At the following website https://www.cellscale.com/products/mcfx/ :

- an introductory video
- all technical info regarding the plate
- mcfx user manual
- mcfx software can be found.

An in-house tray was developed to allow for absorbance and fluorescence readings directly on live cells within the silicone-well plate culturing system. TECAN spark^{R} multimode reader (Tecan group, Männedorf Switzerland) was calibrated to perform measurements right in the center of each well of the silicone plate. Parameters useful for setting up any specific plate reader follow:

The well-to-well spacing and the depth of the wells were measured with the e-drawing program (Fig. **S5 A**) all extra information was obtained from the prompt answers of the cell scale technical support. The measured parameters were used to set up the plate parameters of the multiple-well reader as displayed in Fig. **S5 B** and **C.** During method development, the cell viability and the alkaline phosphatase activity measurements were concurrently performed on and off the silicone plate. The silicone plate measurements were confirmed to reproduce the trend detected on the 96-well plate.

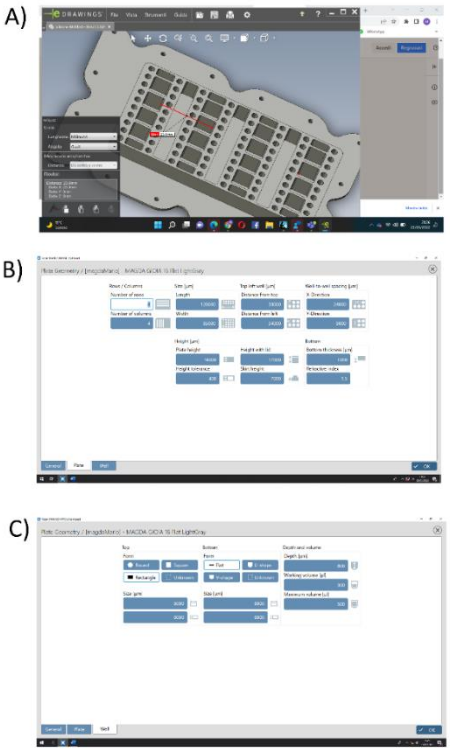

Figure S8. The Parameters employed for setting the coordinate of multiple readers: **A**, the needed measurements were derived from the STEP file of the silicone plate. **B**, plate geometry setting of the silicone plate **C**, plate geometry setting of the wells of the silicone-well plate

Table S1. Densitometric analysis of the clear bands from the gelatinolytic activity in 1Hz and Ctrl cell-conditioned media, ∆indicates the difference between the means (1Hz - Ctrl) (see Figure **6A**).

**MMP gelatinolytic activity**	A.U.
Activity level average of Ctrl samples	34598
Activity level average of 1Hz samples	53387
∆ means (1Hz - Ctrl) ± SEM	18789 ± 1515

Table S2. Cell extracts were or were not harvested 24 h after the mechanical stimulus (1 Hz and Ctrl respectively) and analysed through Western blotting. Filters were probed with specific antibodies for total Akt (60kDa) and GAPDH (37kDa). The densitometric analysis of Akt signals is shown in arbitrary units (see Figure **6B**). ∆indicates the difference between the means (1Hz - Ctrl)**.**

**Western blot densitometric analysis**	A.U.
Akt level of Ctrl	0.6668
Akt level of 1Hz	0.6680
∆ means (1Hz - Ctrl) ± SEM	0.001260 ±0.07732

Supplementary bibliography

8. Alloisio, G. *et al.* Effects of Extracellular Osteoanabolic Agents on the Endogenous Response of Osteoblastic Cells. *Cells* **10**, (2021).

58. Boccafoschi F, Bosetti M, Sandra PM, Leigheb M, Cannas M. Effects of mechanical stress on cell adhesion: a possible mechanism for morphological changes. Cell Adh Migr. 2010 Jan-Mar;4(1):19-25. doi: 10.4161/cam.4.1.9569. Epub 2010 Jan 21. PMID: 19829055; PMCID: PMC2852553.

59.Jung, Y. J. *et al.* Focused low-intensity pulsed ultrasound enhances bone regeneration in rat calvarial bone defect through enhancement of cell proliferation. *Ultrasound Med Biol* **41**, 999–1007 (2015).

60. Nagaraja MP, Jo H. The Role of Mechanical Stimulation in Recovery of Bone Loss-High versus Low Magnitude and Frequency of Force. Life (Basel). 2014 Apr 2;4(2):117-30. doi: 10.3390/life4020117. PMID: 25370188; PMCID: PMC4187165.

25. Papachroni, K. K., Karatzas, D. N., Papavassiliou, K. A., Basdra, E. K. & Papavassiliou, A. G. Mechanotransduction in osteoblast regulation and bone disease. *Trends in Molecular Medicine* **15**, 208–216 (2009).

53. Tran, R. D. H., Morris, T. A., Gonzalez, D., Hetta, A. H. S. H. A. & Grosberg, A. Quantitative Evaluation of Cardiac Cell Interactions and Responses to Cyclic Strain. *Cells* **10**, 3199 (2021).

61. Zhou, J. *et al.* The frequency window effect of sinusoidal electromagnetic fields in promoting osteogenic differentiation and bone formation involves extension of osteoblastic primary cilia and activation of protein kinase A. *Cell Biol Int* **45**, 1685–1697 (2021).

The original blot image and zymography gel presented in Fig. 6 was pasted according to the digital image integrity policy of the journal.

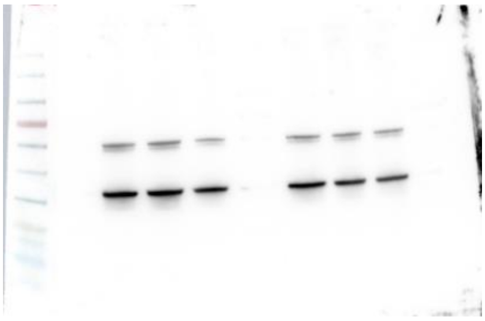

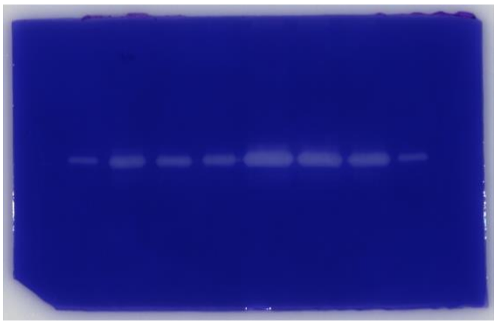

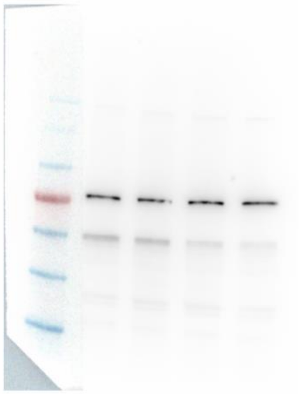

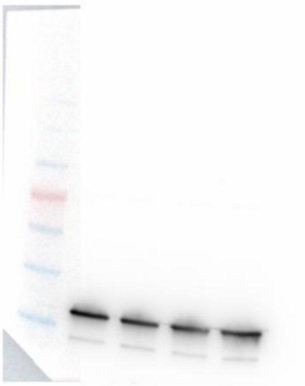

Supplement: Supplementary file 1 [file ijms-24-07686-s001.zip › ijms-2305759-supplementary.pdf]
